# Perception of hypertension management by patients and doctors in Asia: potential to improve blood pressure control

**DOI:** 10.1186/s12930-015-0018-3

**Published:** 2015-02-11

**Authors:** Abdul Rashid Abdul Rahman, Ji-Guang Wang, Gary Mak Yiu Kwong, Dante D Morales, Piyamitr Sritara, Renan Sukmawan, on behalf of all members of the Asian Cardiovascular Expert Forum Committee

**Affiliations:** An Nur Specialist Hospital, No. 19 Medan Pusat Bandar 1 Section 9, 436550 Bandar Baru Bangi, Kajang Malaysia; Centre for Epidemiological Studies and Clinical Trials, The Shanghai Institute of Hypertension, Ruijin Hospital, Shanghai Jiaotong University School of Medicine, Shanghai, China; Pro-Cardio Heart Disease & Stroke Prevention Centre, Tsim Sha Tsui, Hong Kong; Department of Internal Medicine and Cardiology, Manila Doctors Hospital, Manila, Philippines; Praram 9 Hospital, Bangkok, Thailand; Department of Cardiology and Vascular Medicine, University of Indonesia, Harapan Kita National Cardiovascular Center, Jakarta, Indonesia

**Keywords:** Attitude to health, Hypertension, Hong Kong, Indonesia, Malaysia, Philippines, South Korea, Taiwan, Thailand, Qualitative research

## Abstract

**Background:**

Hypertension is one of the world’s most common health conditions and is a leading risk factor for mortality. Although blood pressure can be modified, there is a large proportion of patients whose blood pressure remains uncontrolled. The aim of this study, termed Edvantage 360°, was to gain a deeper understanding of hypertension management in Asia from the perspective of patients and doctors, and to propose strategies to improve blood pressure control.

**Methods:**

Conducted in Hong Kong, Indonesia, Malaysia, the Philippines, South Korea, Taiwan, and Thailand, Edvantage 360° was a mixed-methods observational study that used both qualitative and quantitative elements: qualitative interviews and focus groups with patients (N = 110), quantitative interviews with patients (N = 709), and qualitative interviews with doctors (N = 85).

**Results:**

This study found that, although there is good understanding of the causes and consequences of hypertension among Asian patients, there is a lack of urgency to control blood pressure. Doctors and patients have different expectations of each other and a divergent view on what constitutes successful hypertension management. We also identified a fundamental gap between the beliefs of doctors and patients as to who should be most responsible for the patients’ hypertension management. In addition, because patients find it difficult to comply with lifestyle modifications (often because of a decreased understanding of the changes required), adherence to medication regimens may be less of a limiting factor than doctors believe.

**Conclusions:**

Doctors may provide better care by aligning with their patients on a common understanding of successful hypertension management. Doctors may also find it helpful to provide a more personalized explanation of any needed lifestyle modifications. The willingness of the doctor to adjust their patient interaction style to form a ‘doctor-patient team’ is important. In addition, we recommend that doctors should not attribute ineffectiveness of the treatment plan to patient non-adherence to medications, but rather adjust the medication regimen as needed.

**Electronic supplementary material:**

The online version of this article (doi:10.1186/s12930-015-0018-3) contains supplementary material, which is available to authorized users.

## Background

Hypertension (high blood pressure) is one of the world’s most common chronic health conditions [1] and is a leading risk factor for mortality and disease burden [2]. The prevalence of hypertension in Asia is similar to the prevalence in Western countries and ranges from 23% to 41% among men and from 11% to 34% among women [3]. It has been estimated that in Asia up to 66% of deaths from cardiovascular disease, including stroke, may be attributable to hypertension [3].

Despite these statistics, blood pressure can be modified and there are clear treatment guidelines available to help doctors and their patients control hypertension (for example, at the time of this study, JNC 7 [4], ESH-ESC 2007 [5] and NICE 2011 [6]). The recommended management for hypertension includes modification of a variety of lifestyle factors, such as reducing salt intake, obesity, physical inactivity, alcohol intake, and psychological stress, and the use of a range of effective antihypertensive medications. However, despite these recommendations and available therapies, the proportion of patients whose blood pressure remains uncontrolled remains relatively high [7] and may be up to 54% of diagnosed Caucasian patients [8]. For Asian patients, the proportion of patients whose blood pressure remains uncontrolled is even higher than 70% [9–12].

To understand why hypertension is so difficult to control, it may be of benefit to gain an understanding of the patients’ perspectives of their condition. In addition, given that a patient’s perspective is often very different from that of his or her doctor [13], a better understanding of the differences might offer further insight into how doctors can help patients control hypertension. Although patient perspectives on hypertension have been well studied in Western countries [13], little is known about how hypertension is perceived by patients from Asia, where racial and societal factors may lead to notable differences from Western countries.

Therefore, the aim of this study, termed Edvantage 360°, was to gain a deeper understanding of the management of hypertension in Asia from the perspective of both patients and doctors. To gain this understanding, the study set out (i) to qualitatively assess - using beliefs, attitudes, behaviors, and opinions - how patients with hypertension in Asia (Hong Kong, Indonesia, Malaysia, the Philippines, South Korea, Taiwan, Thailand) perceive this condition and (ii) to compare the perspectives of patients to those of the treating doctors. Because the perception of hypertension among Asians has not been clearly elucidated, this study could provide relevant and valuable information to Asian doctors who treat patients with hypertension.

## Methods

### Study design

Edvantage 360° was a mixed-methods observational study that used both qualitative and quantitative elements. There were (a) qualitative interviews and focus groups with patients (b) quantitative interviews of patients, and (c) qualitative interviews of doctors. The questionnaires and discussion guides for each element of the study are included as supplementary information. (Additional file 1, Edvantage Questionnaires and Guides). The Edvantage 360° study was conducted in accordance with the ESOMAR (European Society for Opinion and Marketing Research; www.esomar.org) Code of Conduct, and all patients and doctors provided written informed consent. The study was conducted from July to August 2012 in Hong Kong, Indonesia, Malaysia, the Philippines, South Korea, Taiwan, and Thailand (with pilot studies conducted in Singapore).

### Study population

Patients were recruited using face-to-face or phone methods via (i) doctor recruitment, where doctors invited 3 consecutive patients to participate, (ii) snowballing, where patients were asked if they knew other patients diagnosed with hypertension, and (iii) hospital or clinic intercept, where patients were intercepted as they left the hospital or clinic. Patients were selected using quota (non-probability) sampling. The population was segmented into mutually exclusive sub-groups (e.g., by country and/or region) and then patients were selected based on pre-defined inclusion criteria and invited to participate.

The inclusion criteria for patients were age 35 to 65 years, diagnosis of hypertension for at least 12 months, systolic blood pressure at least 140 mmHg (or 130 mmHg for patients with diabetes), and currently on prescription medication for hypertension for at least 6 months (while having never deliberately stopped taking their medication). The study noted that half of the patients were to be on an angiotensin II receptor blocker (to ensure that the study was conducted where there had been adoption of current hypertension management), and it also defined a minimum household income level for each country or region (to ensure that all included patients had access to medication). During screening, some patients declined to participate (the most common reason being that the questions were too complicated), but there were very few dropouts after the study began.

Doctors from both the public and private sectors were recruited from a list of potential doctors in each city, compiled by consulting medical societies, hospital and clinic websites, and public telephone directories. Doctors were randomly selected from the list, screened, and invited to participate. The inclusion criteria for doctors included consulting with at least 80 patients with hypertension per month and prescribing angiotensin receptor blockers to at least 50% of their patients with hypertension.

### Patients: qualitative interviews and focus groups

Qualitative in-home, in-depth, face-to-face interviews were conducted with approximately 15 patients per country or region (18 from Malaysia, 17 from Hong Kong, and 15 each from South Korea, Taiwan, Indonesia, Thailand, and the Philippines), for a total of 110 interviews.

The qualitative interviews (lasting approximately 30 minutes each) were conducted by degree-qualified, professional, experienced, trained interviewers. There were a total of 16, mostly female, interviewers who had no relationship to the patients. During the interviews, which were audio recorded and may also have been documented with field notes, only the interviewer and the patient were present. All patients selected for the qualitative interview completed a video diary and journal to record the impact of, and their feelings towards, hypertension, and a follow-up interview (face-to-face or by telephone for 10 minutes).

The focus group discussions included 3 patients from each country or region. These face-to-face conversations lasted for 90 to 120 minutes at a central location in each country or region. The focus group discussions were conducted by degree-qualified, professional, experienced, trained interviewers. There were a total of 7 interviewers, who were all female and had no relationship to the patients. During the discussions, which were video recorded, other personnel such as project managers may have been present although they did not participate. The interviewers discussed with the patients their understanding of, and their attitudes towards, hypertension, their experiences of consultations with doctors, and their understanding of blood pressure control.

### Patients: quantitative interviews

In addition to the qualitative interviews, approximately 100 patients took part in quantitative face-to-face interviews in each country or region (108 from Malaysia, 101 from Philippines, 100 each from South Korea, Taiwan, Hong Kong, Indonesia, and Thailand), for a total of 709 interviews. The interviews (lasting approximately 20 minutes each) were conducted by professional, trained interviewers. There were a total of 60 interviewers, male and female, who all had no relationship to the patients. During the interviews, which were audio recorded and may also have been documented with field notes, only the interviewer and the patient were present. The interviewers recorded information on patient attitudes towards hypertension, measurement of blood pressure, consultations with doctors, and patient demographics and characteristics.

### Doctors: qualitative interviews

Finally, qualitative in-depth face-to-face interviews were conducted with 12 doctors per country or region (13 doctors from Hong Kong), for a total of 85 individual interviews. Doctors were general practitioners (6 per country or region), or cardiologists (6 or 7 per country or region). The interviews (lasting approximately 45 minutes each) were conducted by degree-qualified, professional, experienced, trained interviewers. There were a total of 16 interviewers, mostly female, who had no relationship to the doctors. The interviewers discussed with the doctors their approach to management of hypertension, their perceptions of uncontrolled hypertension, and their interaction with patients.

### Statistical information

The study was underpinned by content analysis theory and themes were derived from the data. To ensure consistency, data were managed at a central location using IBM SPSS Data Collection v5.6 software.

## Results and discussion

This is the first large-scale study to consider hypertension from the perspective of patients and doctors from around Asia. Patients in this study had been diagnosed with hypertension, and the majority (96%, 678 of 709) had been taking medication for hypertension for at least 12 months. Similar to studies in other areas of the world, and with no major differences among the countries and regions surveyed, we found that patients typically understood both the causes and the potential consequences of hypertension. However, our study was able to (A) highlight a number of factors that describe how hypertension is perceived by patients in Asia, and (B) uncover aspects of management where the expectations of the patient and the doctor are different. Based on this information, we provide recommendations for doctors that could help address blood pressure control in patients from Asia.

### (A) Patient perceptions: living with hypertension

Our study highlighted a number of factors that describe patients’ awareness of hypertension and its complications (including in comparison to other diseases), their acceptance of the diagnosis, and their views on management of the condition, including use of blood pressure monitors at home.

#### (A1) Patient knowledge of hypertension

Patients in this study were aware that the leading causes of high blood pressure included high stress, salt intake, and alcohol consumption; this is similar to patient awareness around the world [13]. Patients also understood that hypertension is a chronic lifelong condition, although it was often regarded as a disease that affects the elderly.

**Figure 1:**
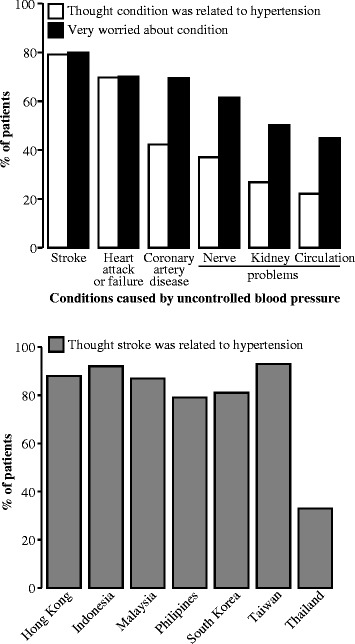
**Patient perceptions of hypertension consequences.** Patients believed that hypertension could lead to severe consequences. Top panel, white bars: patients who thought the condition was related to hypertension (N = 709). Top panel, black bars: of the patients who thought the condition was related to hypertension, patients who were very worried about the condition (stroke N = 577, heart attack or failure N = 563, coronary artery disease N = 357, nerve problems [loss of feeling, numbness] N = 366, kidney problems N = 269, circulation problems [e.g. ulcers] N = 234). Bottom panel, grey bars: patients from each country and/or region who thought stroke was related to hypertension. Of note, patients from Thailand were most concerned about nerve problems.

Patients realized that hypertension could lead to severe consequences such as stroke, paralysis from stroke, and heart attack (Figure 1). The general concern about the potential consequences of hypertension was similar to that observed in other studies [14]. This point was highlighted by patients from Malaysia, Taiwan, Hong Kong, and Philippines who described hypertension in terms such as:

*“the silent killer”, a “time bomb waiting to explode”, or “the traitor in my body”*

The greatest concern among Asian patients was the possibility of stroke (79%, 561/709; of note, a smaller proportion of patients from Thailand were concerned with the risk of stroke [Figure 1]). The fact that stroke was generally the greatest concern may be because the risk of stroke is indeed greater for Asians than for Caucasians [15,16], leading to a high incidence of stroke in Asia [17]. Therefore, stroke, and the associated immobility and family burden, were very prominent in the thoughts of the patients in this study, as illustrated in the following quote:

*“If hypertension is not controlled carefully (it will cause) stroke. I don’t want to end up becoming immobilized.”* (patient from Malaysia).

Despite acknowledgment of the high stroke risk as well as other complications, patients nonetheless (and paradoxically) regarded hypertension as less severe than other medical conditions, although there were differences among countries and regions. The parameters that are related to this view included the perceived ease of blood pressure control and the lack of symptoms of hypertension. These parameters are highlighted by the following quotes:

*Hypertension is “not very severe because I am able to control it.”* (patient from Thailand). *“Rheumatoid arthritis (is more severe than hypertension) because it affects my quality of life.”* (patient from Hong Kong).

#### (A2) Acceptance of receiving the diagnosis of hypertension

When patients were first diagnosed with hypertension they reported feeling worried, anxious, confused, depressed, and/or stressed. To various degrees, patients went through the well-characterized emotional journey from initial feelings of shock to eventual feelings of acceptance (confident, optimistic, hopeful, protected, peaceful, assured [Table 1]). Although patients commonly went on this emotional journey, only 43% (302 of 709) of patients had accepted that hypertension was part of their lives. In addition, the diagnosis of hypertension typically had only a limited effect on the patients’ general mood (only 15% [103 of 709] of patients were affected) or their ability to sleep or work (only 13% [92 of 709] of patients were affected).

**Table 1:** **Steps in the emotional journey of patients after diagnosis with hypertension**

**Step**		**Quote**
Step 1	Shock	*“The nurse shouted ‘How high!’ She said that (my blood pressure was) 200 (systolic). I was so shocked I thought she was lying.*” (patient from Indonesia)
Step 2	Denial	*“At first I didn’t take it very seriously. I simply thought that there has been something wrong with the result.”* (patient from South Korea)
Step 3	Anger	*“Why it happens again? Why me?”* (patient from Hong Kong)
Step 4	Bargaining	*“I wasn’t willing to surrender to the idea of taking medications until the third or fourth doctor I saw.”* (patient from Malaysia)
Step 5	Depression	*“It was depressing and sad back then because you knew you had to make a lot of adjustments.”* (patient from Philippines)
Step 6	Testing	*“There was a period of time when I was really resistant of it. I stopped taking (medication) for a couple of days. My blood pressure went up and down like a yo-yo.”* (patient from Taiwan)
Step 7	Acceptance, passive	“*It is easy for me now. I am not planning for any further effort. I would just take the medication and check for my blood pressure to see if it is within my target level.”* (patient from Taiwan)
	Acceptance, motivated	*“I saw people around me suffering from cerebral infarction or cerebral hemorrhage so I am more concerned but it is not easy to exercise regularly. It is not easy to raise children and have my own time to exercise, so I try to move as much as possible, but it is not as easy as I thought.”* (patient from South Korea)

The limited acceptance of hypertension observed in this study is similar to that reported for patients from Western countries. For example, a study in Finland found that 66% of 1,561 patients had difficulties accepting hypertension, and 63% of patients had a careless attitude towards hypertension [18]. In addition, a study in Italy found that, even though patients were aware that hypertension was a risk factor for stroke, and that stroke could be a consequence of hypertension, they still did not consider hypertension to be a serious health concern [19]. This limited acceptance may be reflective of the patients’ view of hypertension as a “*silent killer”*, because of the lack of overt physical symptoms that patients experience, particularly compared with other medical conditions. This lack of overt physical symptoms may ironically be a negative aspect of this condition, as patients may be able to deny the existence their high blood pressure more easily. In addition, the mental quality of life of patients, which was found to be negatively impacted by simple awareness of hypertension in a Thai population [20], may also influence the motivation level of patients to accept hypertension as part of their lives.

#### (A3) Factors that drive patient management

Our study identified a dynamic balance of issues that motivated or hindered patients during the management of their blood pressure.

Factors that motivated patients to continue to manage their hypertension included issues that seemed to maintain their sense of normalcy. The most common issues included (i) the patients’ hopes to avoid future health problems resulting from their hypertension, (ii) their desires to maintain blood pressure below numerical target levels while also avoiding increases in the dosage or amount of medications, and (iii) their hopes that this condition may have only a minimal impact on their everyday lives. Such motivating factors among patients are highlighted by a quote from a patient from Indonesia:

*“[The] motivation is to get better soon. Recovered, healthier, no more disease.”*

Conversely, patients also noted many aspects of their condition that caused them frustration or anxiety, which all seemed to reflect a notable change in the patients’ viewpoint of being ‘normal’. The most common issues included (i) the need to take medication every day, (ii) the prospect of possible future cardiovascular complications (such as stroke and the potential resultant paralysis), (iii) the need for consistent exercise and diet control, (iv) the idea that hypertension is a chronic condition with no cure, (v) long-term side-effects of their medication, (vi) the less-than-satisfactory visits with their doctor, and (vii) denial of a similar lifestyle as that of their peers (particularly for patients under 50 years).

Although there was a dynamic balance of issues that motivated or hindered patients during the management of their blood pressure, the desire to feel normal was a consistent theme in patient behavior.

#### (A4) Patient self-monitoring of blood pressure

Just over half (57%, 405 of 709) of the patients in this study did not have their own blood pressure monitor, and there were country and/or region-specific differences in the perception among patients of the need to self-monitor blood pressure. The most common reason that patients in this study claimed that they did not own a blood pressure monitor was the belief that blood pressure should be monitored by a doctor (noted by 45% [184 of 408] of respondents). However, the proportion of patients varied significantly among countries and regions, from only 9% (6 of 64) of patients in Philippines and 17% (2 of 12) of patients in Hong Kong to 79% (11 of 14) of patients in Taiwan. Another reason that patients gave for not owning a blood pressure monitor, which was possibly important, was its cost; noted by 17% (71 of 408) of patients overall (and by as high as 45% [29 of 64] of patients in Philippines). Moreover, 42% (5 of 12) of patients from Hong Kong believed that the process of measuring blood pressure was too complex for them to do it by themselves.

Reluctance by patients to self-monitor blood pressure was evident by the negative perceptions of blood pressure monitoring. Patients (i) reported they could supposedly ‘feel’ if their blood pressure was controlled, (ii) believed blood pressure readings could be inaccurate because of stress and fluctuations in blood pressure, (iii) did not feel confident or qualified to take their own blood pressure, and (iv) felt that a monitor at home would be a constant reminder of their condition. Even among patients who owned a blood pressure monitor, use of the monitor typically declined after initial diagnosis and, often, the monitor was only used either when the patient experienced some type of physical symptoms, or the day before they were scheduled to visit their doctor. This attitude is highlighted by a quote from a patient from Hong Kong:

*“I [measured it every day] for a week and then I stopped; I think it’s no use.”*

Although patients in our study were reluctant to self-monitor blood pressure, self-monitoring of blood pressure has been shown to play an important part in hypertension management [21]. The advantages of patient measurement of blood pressure include a potentially enhanced relationship with their doctor, although doctors must be willing to participate and provide guidance and support to patients [22].

### (B) Differences between the perceptions of patients and doctors

This study also revealed many differences between the perceptions of Asian patients and doctors regarding blood pressure management (Table 2). In particular, differences were noted with regard to interaction at clinic visits, the definition of good blood pressure control, and the methods to manage hypertension.

**Table 2:** **Summary of the differences between the perceptions of patients and doctors for management of hypertension**

**Perception**	**Patients**	**Doctors**
Most important factor controlling blood pressure	Medication	Patient behavior
Believe patients are adherent to medication	Yes	No
Believe lifestyle modifications are important	No	Yes
Believe treatment is successful if….	Adherent to medication, no symptoms, attending appointments	Target blood pressure reached
Blood pressure for concern	160/100 mmHg	140/90 mmHg
Expectation of consultations	Expected doctors to take initiative	Expected patients to take initiative
Belief about each other	Doctors not concerned about them	Patients did not accept their role in management of the disease

#### (B1) The perception of clinic visit interactions

From this study, it is clear that doctors and patients had different expectations for clinic visits. In accordance with guidance for management of hypertension [4–6], doctors expected patients to take the initiative to improve their lifestyles. If a patient had uncontrolled blood pressure, despite being prescribed medical therapy, doctors often commented generally on making further lifestyle improvements and maintaining adherence to medications. A typical quote would be:

*“Please take your medication regularly and control your diet as much as you can.”* (cardiologist from Thailand).

However, while doctors noted that patients often expressed concern about lack of blood pressure control and promised to make changes to meet target blood pressure goals, they believed that patients were unmotivated and put in little effort to manage their hypertension. Despite being apologetic to the doctor, patients also made excuses for their unhealthy lifestyles and lack of adherence to their doctors’ guidance, which they attributed to external factors such as work schedules, etc. Of note, doctors were more sympathetic to older patients (who may have other medical conditions) than to younger patients, who they believed should put in more effort to manage their hypertension. This is highlighted by a quote from a general practitioner from South Korea:

*“I think you have problems with your lifestyle, you are a business man and I think you enjoy eating too much fatty meal and drinking.”*

Patients, on the other hand, stated that they expected doctors to take the initiative and ensure that their hypertension was well managed. Patients found consultation visits too brief, with one-way communication, and as a result would ask few questions. Patients also believed that consultations were not ‘value-added’; the doctor provided a general, qualitative assessment of blood pressure and reminders to take medications, but they usually did not provide patients with specific helpful information. If the doctors did provide advice, it was on generic lifestyle improvements. Doctors were usually not sympathetic towards the patients’ individual situations, their specific concerns, nor their difficulties in managing their own blood pressure. This is highlighted in a quote from a patient from Malaysia:

*“Most of the time we would usually have a long waiting time, [and] we often have a long list of questions in mind to ask the doctor. When we finally get to see the doctor we’re simply told our condition is ‘fine’, and they can’t wait to get rid of us.”*

Therefore, although doctors and patients had a similar understanding of how blood pressure could be controlled, they had very different expectations of each other. Patients’ beliefs that doctors should be more engaging and proactive and that doctors do not seem to be concerned about them sets off a cycle of mistrust between doctors and patients. On the other hand, doctors cited how they have very little time and resources – and (often) desire – to motivate patients to control their hypertension, at least partially because doctors generally believed that patients did not truly accept their responsibility to manage their own condition.

### Recommendation 1 for doctors: consultations

We recommend that doctors show a more optimistic attitude toward their patients and provide greater empathy and individual support (Table 3). This approach may improve patient adherence to the doctor’s advice to a greater degree than providing general recommendations. Doctors’ perceptions of hypertension can influence the support they give their patients. For example, doctors who are motivated to manage hypertension with a confident and optimistic approach are more successful at controlling blood pressure [23].

**Table 3:** **Summary of recommendations for doctors treating patients with hypertension**

**No.**	**recommendations**
1	That doctors show a more optimistic attitude toward their patients and provide greater empathy and individual support
2	That doctors continue to emphasize to patients the importance of reaching target blood pressure values
3	That doctors continue to emphasize the important of lifestyle modifications to patients and to explain to them that lifestyle changes can lead to improved outcomes

In addition, one of the main barriers to optimal control of hypertension is poor communication between doctors and patients [24]. This poor communication can be exacerbated by a lack of doctor training and skills in lifestyle counselling and the severe time constraints that doctors work under [24]. Therefore, by using counselling and engagement skills, doctors may more effectively and efficiently care for their patients.

#### (B2) The perception of how to define successful hypertension management

Doctors had an objective perspective on the definition of successful management of hypertension; they considered the key measure to be whether blood pressure targets were reached. Further, doctors believed that patient behavior was primarily responsible for the uncontrolled blood pressure and unsuccessful hypertension management. Doctors correlated factors such as poor patient adherence to the medications and poor lifestyle choices (including overeating, high salt intake, lack of exercise, high alcohol consumption, and smoking) with uncontrolled blood pressure. In particular, doctors tended to blame obese patients, as highlighted by a quote from a cardiologist from Thailand:

*“For overweight patients it may be difficult for them to control weight as they eat a lot of salty food. They don’t control food and eat salty food [and] do not exercise.”*

In contrast, patients had a more subjective perspective on successful management of hypertension. Patients perceived that successful hypertension management was not defined by reaching a target blood pressure, but rather, involved taking their medication regularly, attending scheduled check-ups with their doctor and having their blood pressure measured, and experiencing a lack of symptoms (such as dizziness or headaches). Most patients were not concerned with reaching any target blood pressure goals and accepted fluctuations in blood pressure.

When doctors and patients did consider target blood pressure levels, the specific numbers for acceptable control often differed. Doctors described a patient as having uncontrolled hypertension in accordance with common medical practice and recommended guidelines when blood pressure was greater than 140/90 mmHg. Furthermore, doctors expressed appropriate concern for patients with uncontrolled hypertension because of the increased risk of severe complications and/or death, as noted by a cardiologist from Malaysia:

*“Of course I’m very concerned because it increases the risk of you getting a stroke or heart attack.”*

**Figure 2:**
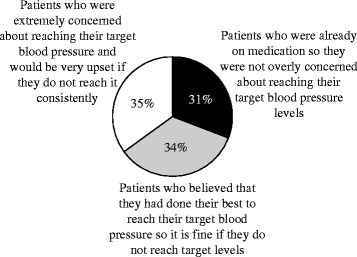
**Patient beliefs regarding target blood pressure.** Patients were generally only moderately concerned about reaching target blood pressure levels, although patient responses varied (N = 709).

In contrast, patients were typically not concerned with their hypertension until their blood pressure was over 160/100 mmHg. In general, patients were only moderately concerned about reaching numerical target blood pressure levels, although patient responses varied (Figure 2). Again, patients believed that their hypertension was controlled if they were modifying their lifestyle as much as possible, if they were taking their medications daily, and if they had no symptoms. Some patients felt that there should be different, individual target levels for each person. This attitude is highlighted by a quote by a patient from Taiwan:

“*Everyone has difference when it comes to tolerance [of blood pressure levels], maybe her tolerance is higher than mine. If my blood pressure goes above the number [160] then my body would start experiencing warning symptoms.”*

Although doctors believed that patient behavior was the key factor contributing to uncontrolled hypertension, other factors were also noted including the presence of other medical conditions and inadequate or ineffective medication (particularly noted by doctors from South Korea, Philippines, and Malaysia).

There may also be doctor-related factors that contribute to uncontrolled blood pressure; for example, it is possible that doctors had clinical inertia: a failure to initiate or appropriately intensify medication therapy for patients with uncontrolled hypertension [24]. For example, in a study in the United States, 33% of 1,200 doctors would not intensify medication therapy for patients with a systolic blood pressure of 158 mmHg [25], which is inconsistent with the recommended level of 140 mmHg. In addition, one year after the Japanese guidelines for treating hypertension were released, only 19% of 446 doctors in Japan would initiate medication at 140 / 90 mmHg as recommended [26]. Other studies have shown that a major limitation to treating patients is that doctors are of the opinion that a lack of medication adherence by patients is the greatest barrier to controlling hypertension [27].

### Recommendation 2 for doctors: target blood pressure

We recommend that doctors continue to emphasize to patients the importance of reaching target blood pressure values (particularly as guidelines are increasingly based upon accumulating clinical evidence; Table 3). We believe that doctors should clearly explain to their patients the specific risks of the consequences of high blood pressure, and that doctors should consistently remind patients that a lack of symptoms does not imply a lack of risk. Patients need to be convinced of the risks of hypertension to ensure they stay motivated to control their blood pressure within the range that clinical studies suggest is the most beneficial for the patients’ long-term health. It may also be important that, given patients view adherence to the prescribed regimen as successful hypertension management, doctors ensure the treatments and doses they are prescribing for their patients are effective.

### (B3) The perception of methods to achieve blood pressure control

To control blood pressure, doctors expected patients to make lifestyle changes including losing weight, controlling their diet, and exercising, as well as take their prescribed medications every day. Doctors stated that the most important factor to improve blood pressure control was the attitude and behavior of their patients. As noted by a cardiologist from the Philippines:

*“Uncontrolled hypertension is usually a consequence of a few patient factors.”*

To some extent, patients also believed they were successfully managing their hypertension (i) if they made an effort to change their lifestyle, at least to some extent, and (ii) if they adhered to medication advice.

**Figure 3:**
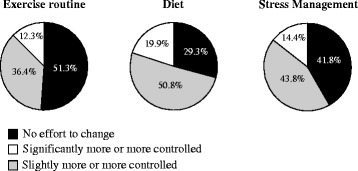
**Lifestyle changes to control hypertension.** Many patients did not change their exercise regimen, diet, or stress management (N = 709).

Similar to findings from studies in other countries [14], this study found that many patients did not adhere to the suggested lifestyle modifications. Specifically, many patients did not change their diet, manage their stress, or follow an exercise regimen (Figure 3). The two main reasons why patients did not improve their lifestyle were because patients did not understand why they needed to change, and because patients did not understand specifically how to make changes. For example, changes in diet were often based on a nonspecific suggestion of a healthy diet rather than specific food and nutrition advice provided by health professionals. In addition, when patients tried to exercise, it often occurred irregularly, and only weekly or even monthly: and there was a group of patients who justified their lack of exercise, as highlighted by this quote:

*“Sometimes, I just feel exhausted after work, I don’t feel like doing anything….or if I go to exercise, I’ll feel very tired at work the next day.”* (patient from Hong Kong)

Further, patients in our study implied that they were less diligent at making lifestyle changes because they believed that hypertension medication usage was the primary way to control hypertension, much more so than lifestyle factors.

With regards to adherence, patients understood that they needed to take their medication and, therefore, were generally adherent to their hypertension medication. In this study, 82% (578 of 709) of patients believed themselves to be highly adherent to their medications, and 68% (484 of 709) of patients reported that they always refilled their prescription before the previous one ran out. However, this was in contrast to the view doctors had on adherence, where estimates of patient medication regimen adherence rates varied widely (from 15% to 100%).

### Recommendation 3 doctors: achieving blood pressure control

We recommend that doctors continue to emphasize the importance of lifestyle modifications to patients and to explain to them that lifestyle changes can lead to improved outcomes (Table 3). Doctors should consider the patient’s emotional stage in their acceptance of hypertension when providing recommendations and note that even patients who are accepting of their diagnosis are still often reluctant to follow through on lifestyle advice. In addition, patient education should include specific details on exactly how to modify diet and exercise, ideally tailored to the patient’s personal situation. However, doctors should always consider that patients may not follow advice on lifestyle modification, and therefore should ensure that the prescribed medications are sufficient to control blood pressure. If a given prescription is not effective, a change in regimen or medication should be considered.

There is no evidence that the fundamental information included in treatment guidelines for hypertension and associated educational interventions needs to be tailored to cultural or ethnic groups [13,28], and there is abundant information available to doctors to assist in improving the management of their patients’ hypertension. In 2008, expert recommendations for improving management of hypertension in Asia were published [28], and these guidelines included suggestions such as patient education, an effective doctor-patient relationship, and doctor education. The findings from our study support these recommendations.

### Strengths and limitations

The main strengths of our study are the multi-element design that considered the opinions of a large number of patients from many Asian countries and regions, and the direct comparison of the perspectives of doctors and patients. However, patients who are willing to participate in interviews and focus groups may not be representative of the general population. We do not believe there is any reason our findings would not be relevant to Asian countries and regions in general, including those not represented in this study, as well as to many Western countries. When applying the findings from this study to other populations, it is important to note that the patients in this study were mostly from urban areas and only included patients who had not deliberately stopped taking their hypertension medications. However, adherence with medications was not measured in this study, and it is possible that patients who were willing to participate may not have acknowledged whether they had deliberately stopped taking their medication. In addition, as hypertension medication nonadherence rates among Asian patients may be as high as 56% [29], some findings from our study are only relevant to the half of the hypertensive population who are willing to take their medications.

## Conclusions

Our study revealed many differences between the perceptions of patients and the perceptions of doctors with regard to managing hypertension in Asia. Of note, doctors and patients had different expectations of each other, and we were able to illustrate how these differences may contribute to a lack of blood pressure control in many patients.

Our study found that patients were generally aware of the causes and consequences of hypertension, and the possibility of stroke (which may lead to immobility and family burden) was their greatest fear. Nonetheless, patients generally believed that their hypertension was not of immediate concern (possibly due to the lack of physical symptoms), and this reduced their acceptance of the diagnosis and their sense of urgency to control their blood pressure. We identified many reasons why blood pressure control may be suboptimal, including patients’ desire to maintain a sense of normalcy, patients’ disinclination to monitor their own blood pressure at home, a lack of understanding of exactly what lifestyle changes to make and how to make them, and patients’ measurement of successful hypertension management by their attempts to manage the condition rather than their actual blood pressure. We also found that patient adherence to medication may not be as limiting as doctors might believe (although adherence was not measured in this study).

Furthermore, there is a gap between the beliefs of doctors and patients as to who should be most responsible for the patient’s hypertension management, and what defines successful hypertension management. Although there is agreement by patients and doctors that both lifestyle modifications and medication regimens can help control blood pressure, medication is often more practical to implement and easier for patients than lifestyle changes.

Finally, we suggest that the most important factor in convincing patients to manage their hypertension is their relationship with their doctor. Patients should be convinced by their doctor of the risks associated with hypertension and the importance of reaching recommended blood pressure targets. It is essential that doctors take steps to educate patients about lifestyle modifications, and that they accept that patients are generally adherent to medications. However, it is imperative that the prescribed medication regimen be an effective one.

In conclusion, we have proposed some improved points of communication between doctors and patients (Table 3), and it is possible that such recommendations could help improve the management of hypertension in Asia. The crux of these recommendations is that doctors should accept greater responsibility for their role in assisting patients to manage their hypertension. Successful hypertension management will rely on the doctor’s willingness to adjust their patient interaction style as necessary to form a ‘doctor-patient team’.

## Additional file

Additional file 1:
**Edvantage Questionnaires and Guides.**
