# Supplementary material for: Perception of hypertension management by patients and doctors in Asia: potential to improve blood pressure control
Source: Asia Pac Fam Med. 2015 Feb 11;14(1):2. doi: 10.1186/s12930-015-0018-3 (PMC4344743; doi:10.1186/s12930-015-0018-3)
Supplement: Additional file 1: — Edvantage Questionnaires and Guides. [file 12930_2015_18_MOESM1_ESM.pdf]

Study No: 1348
Study Name: **Project Edvantage – Patient Quant**
Date: **8 June 2012**

This questionnaire remains the property of:
The Research Partnership Ltd
42a Club Street, Singapore 069420
Tel: +65 6 222 4646

**Malaysia**	**1**
**S. Korea**	**2**
**Taiwan**	**3**
**Hong Kong**	**4**
**Indonesia**	**5**
**Thailand**	**6**
**Philippines**	**7**

Respondent number: ☐☐☐

PATIENT SCREENER – QUANTITATIVE

This research is conducted under a code of conduct and is sponsored by a pharmaceutical company

Recruiter to read out:
Good morning / afternoon, my name is *[INSERT NAME]* calling from The Research Partnership a medical market research company. We are currently conducting a market research study in which we will be interviewing people to understand how they get information about healthcare. The interview will last approximately 30 minutes~~, and the focus group which will last around 2 hours, occurring on another day.~~ We would like to offer you *[INSERT INCENTIVE AMOUNT]* to thank you for your help and time.

Would you be willing to take part in this study? Firstly, I need to ask you some questions to ensure that the study is appropriate for you.

Note to recruiter

S1. Do you personally suffer from any of the following health conditions (please check all that apply)? Any other conditions? (read out)

S2. For those suffered conditions, which of them are you currently receiving treatment for? (read out)

Logic Check: Ask S2 only for attributes selected in S1. S2≤S1.

	**S1.**	**S2.**
Coronary artery disease	**1**	**1**
Stroke	**2**	**2**
High blood pressure	**3**	**3**
Chronic Kidney Disease	**4**	**4**

CLOSE IF CODE 3 NOT SELECTED IN S1 AND S2

Dyslipidaemia/ High blood cholesterol	**5**	**5**
Arrhythmia	**6**	**6**
Chronic heart failure	**7**	**7**
Diabetes - insulin dependent	**8**	**8**
Diabetes - non-insulin dependent	**9**	**9**
None of the above/ No	**99**	**99**
Other specify:		

Note to recruiter

S3. **ASK IF CODE 3 SELECTED IN S1 & S2**

SINGLE ANSWER

Please tell me which of the following people confirmed that you have high blood pressure? (Read out)

GPs 1
Cardiologists.................................. 2
Pharmacists.................................... 3
Nephrologists 4
Traditional medicinal hall............. 5
None of the above 6
Other specialists............................ 7

CLOSE IF CODE 3, 5 or 6

S4. **ASK IF CODE 3 SELECTED IN S1 & S2**

How long ago were you **diagnosed** with high blood pressure?

[ ][ ] Years [ ][ ] months

QUOTA

All patients must be diagnosed with HTN for at least a year

Category	Sample
**Newly diagnosed:** **12~23 months**	**50% of Sample size**

CHECK QUOTA

© The Research Partnership

(1 year to 1 year 11 months)	
Advanced HTN patients:<br>24 months and above<br>(2 years and above)	50% of Sample size

S5. *SINGLE ANSWER*

Which of the following statements best applies to your current treatment for high blood pressure? (read out)

Note: Prescription treatment refers to medication prescribed by doctors/ healthcare professionals NOT including Pharmacists

Currently on **prescription treatment**.........1

Previously on **prescription treatment** but has since stopped......2 **CLOSE IF CODE 2 OR 3**

Has never been on any prescription treatment.......3

S6. **ASK IF CODE 1 SELECTED IN S5**

How long have you been on prescription medication?

[ ][ ] Years [ ][ ] months

CHECK QUOTA

QUOTA

All patients must be on prescription treatment for at least 6 months

S7. *SINGLE ANSWER*

Have you ever stopped taking your prescription treatment for high blood pressure due to any reasons in the past **<insert length of time mentioned in S6>?**

Yes.........1

No......2 **CLOSE IF CODE 1**

S8. *Please share with me the medications you are currently taking for your hypertension?*

Category	Brand name	Code
See brand list		
	Others specify:	

QUOTA

At least 50% of the sample size need to be currently on an ARB (Category) drug

CHECK QUOTA

S9. *SINGLE ANSWER*

Please share with me your last BP (blood pressure) reading at the clinic/ at home?

	Reading	Quota
Systolic	mmHG	**Range: 90~230 mmHG**<br><br>All respondents must have a systolic reading of **≥140 mmHG**<br><br>**IF CODE 8 OR 9 IN S2:** a systolic reading of **≥130 mmHG is acceptable**
Diastolic	mmHG	**Range: 60~140 mmHG**

CHECK QUOTA

S10. *SINGLE ANSWER*

Taking into account all aspects of dealing with your condition (diet and exercise, taking your medication, etc), how successful do you think you are at managing your condition, in general? (read out)

Very successful at managing my high blood pressure	***1***
Quite successful at managing my high blood pressure, but with room for improvement	***2***
Sometimes successful and sometimes unsuccessful at managing my high blood pressure	***3***
A little unsuccessful at managing my high blood pressure, but it could be worse	***4***
Very unsuccessful at managing my high blood pressure	***5***

RECORD ONLY

S11. On average, what is your monthly household income? (All except Indonesia)

On average, what is your monthly household expenditure? (Indonesia only)

Income range (grid below) ....

S12. Please tell me your current age?

__________ years

QUOTA

		All countries
**Quant IDIs**	***SC***	***N=100***
Below age 35	*1*	***Close***
Age between 35-45	*2*	***N=30***
Age between 46-55	*3*	***N=40***
Age between 56-65	*4*	***N=30***
Above age 65	*5*	***Close***

S13. Record Race of respondent: (Malaysia only)

Malay 1

Chinese 2

Indian 3

Other.. 4

S14. Record Gender of respondent

Male..1

Female 2

QUOTA

Sample size: Male (50%): Female (50%)

S15. What was your highest educational level achieved?

RECORD ONLY

Less than high school	1
High school graduate	2
Technical / vocation institute	3
College/ under-graduate	4
Degree holder	5
Post-graduate or higher	6
Decline to answer	7
Other, please specify	

S16. Which of the following best describes your present occupation?

RECORD ONLY	Single Code
Owner/Partner	**1**
Senior management (CEO/Chairman/President/Managing Director/Vice President/Director/General Manager/Department Head)	**2**
Professional (eg, doctor, lawyer, engineer)	**3**
Middle Manager	**4**
Executive/Officer/Supervisor	**5**
Technician	**6**
Housewife	**7**
Full-time student	**8**
Retired	**9**
Unemployed	**10**
Other (please specify):____________________________	
Refused	**99**

S17. Are you in any way employed, affiliated or have a close friend or family member working in the following industry:

Pharmaceuticals ....X **CLOSE**

Healthcare ....X **CLOSE**

Marketing/PR....................................X **CLOSE**

Advertising..X **CLOSE**

Other...1

S18. Have you taken part in any market research relating to high blood pressure in the last 3 months?

Yes ....X **CLOSE**

No 2

S19. For this research, the interview may be audio, video-taped and viewed by clients for analysis purposes, however your personal details will remain strictly confidential, as the information you provide will be reported at a country rather than individual level. Do you agree to be audio, video-taped and viewed by clients under these conditions?

Yes 1

No X

S20. Would you be willing for the audio/ video taped interview to be shared with our client? This material will be confidential and would be used for internal market research purposes only.

Yes 1

No X

CLOSE

**What is your monthly household income? Malaysia**		
Below RM 1,500	**1**	**CLOSE**
RM 1,500 to 2,499	**2**	**RECORD**
RM 2,500 to 3,999	**3**	**RECORD**
RM 4,000 to 5,999	**4**	**RECORD**
RM 6,000 to 7,999	**5**	**RECORD**
RM 8,000 and above	**6**	**RECORD**
**What is your monthly household income? S. Korea**		
Below 3,000,000 Won	**11**	**CLOSE**
3,000,000 Won to 3,499,999 Won	**12**	**RECORD**
3,500,000 Won to 3,999,999 Won	**13**	**RECORD**
4,000,000 Won to 4,999,999 Won	**14**	**RECORD**
5,000,000 Won to 5,999,999 Won	**15**	**RECORD**
6,000,000 Won and above	**16**	**RECORD**
**What is your monthly household income? Taiwan**		
Below NT$35,000	**21**	**CLOSE**
NT$35,001 to NT$49,999	**22**	**RECORD**
NT$50,000 to NT$100,000	**23**	**RECORD**
NT$100,001 to NT 149,999	**24**	**RECORD**
NT$150,000 to NT$199,999	**25**	**RECORD**
Above NT$200,000	**26**	**RECORD**
**What is your monthly household income? Hong Kong**		
Below HKD$10,000	**31**	**CLOSE**
HKD$10,000 to NT$19,999	**32**	**RECORD**
HKD$20,000 to NT$29,999	**33**	**RECORD**
HKD$30,000 to NT$39,999	**34**	**RECORD**
HKD$40,000 to NT$49,999	**35**	**RECORD**
Above HKD$50,000	**36**	**RECORD**
**What is your monthly household expenditure? Indonesia**		
E : less than Rp 700,000	**41**	**CLOSE**

D : Rp 700,001 - 1,000,000	**42**	**RECORD**
C2 : Rp 1,000,001 - 1,500,000	**43**	**RECORD**
C1 : Rp 1,500,001 - 2,000,000	**44**	**RECORD**
B : Rp 2,000,001 - 3,000,000	**45**	**RECORD**
A : above Rp 3,000,000	**46**	**RECORD**
**What is your monthly household income? Thailand**		
Below 15,000 Baht	**51**	**CLOSE**
15,000 to 34,999 Baht	**52**	**RECORD**
35,000 to 79,999 Baht	**53**	**RECORD**
80,000 to 99,999 Baht	**54**	**RECORD**
100,000 to 119,999 Baht	**55**	**RECORD**
120,000 Baht and above	**56**	**RECORD**
**What is your monthly household income? Philippines**		
Below PHP 10,000	**61**	**CLOSE**
PHP 10,000 to 29,999	**62**	**RECORD**
PHP 30,000 to 54,999	**63**	**RECORD**
PHP 55,000 to 69,999	**64**	**RECORD**
PHP 70,000 to 89,999	**65**	**RECORD**
PHP 90,000 and above	**66**	**RECORD**

Quota details

Countries	Quantitative IDIs	Total
All countries	100 HTN Patients	**100**

 © The Research Partnership

IMPORTANT NOTE:

- **Please complete the screener for all respondents, only terminate disqualified respondents at the end of the interview**
- **All respondents need to present their medication (previous and currently used) for verification while conducting the interview**

**Respondent contact details and time of interview**	
**Name:**	
**Address:**	
**Phone number:**	
**Email address:**	
**Interview date:**	
**Interview time:**	

1348 Edvantage Patient Quantitative Research
Questionnaire

Rotation

**A**

RESPONDENT DETAILS

Respondent ID no. :

Name of Respondent : ____________________

Address : ____________________

____

Postal Code : ____________ Telephone : ____________

Date of Interview ____________ Length of Interview ____________

Time Started ____________ Time Ended ____________

FIELDWORK RECORD

Interviewer's Name : ____________ Interviewer's ID : ____________

Coder's Name ____________ Validator's Name ____________

I hereby declare that the interview recorded in this Questionnaire was conducted strictly according to instructions specified in the Questionnaire as well as given in the briefing and/or interviewer guide and ICC/ESOMAR International Code. I further declare that the responses recorded in this Questionnaire will be routinely verified with respondents and only considered as accepted after verification. The Research Partnership reserves the right not to pay for any work done if it is deemed not to our satisfaction.

Signature: ____________ **Date:** ____________

Introduction:

Thank you for agreeing to take part in this survey.

The survey will take approximately 20 minutes to complete.

We will be asking some questions about you and your health, please be assured that all your responses will remain confidential and non-attributable.

Attitudes towards Hypertension

Q1. (a) Thinking about how you feel when you were **first diagnosed with hypertension**, please select any of the words below which describe how you feel **then** about your condition. **Interviewer: Show list of emotions to respondent**

(b) Thinking about how you **feel now** about hypertension, please select any of the words below which describe how you feel now about your condition. **Interviewer: Show list of emotions to respondent**

Multi-code per column

SHOWCARD + ROTATE ATTRIBUTES

	A: Then	*B: Now*
Hopeful	*1*	*1*
Confused	*2*	*2*
Depressed	*3*	*3*
Safe	*4*	*4*
Lonely	*5*	*5*
Helpless	*6*	*6*
Frustrated	*7*	*7*
Pessimistic	*8*	*8*
Inspired	*9*	*9*
Protected	*10*	*10*
Confident	*11*	*11*
Peaceful	*12*	*12*
Cared for	*13*	*13*
Neglected	*14*	*14*
Vulnerable	*15*	*15*
Excited	*16*	*16*
Anxious	*17*	*17*
Motivated	*18*	*18*
Informed	*19*	*19*
Optimistic	*20*	*20*
Assured	*21*	*21*
Tired	*22*	*22*
Relieved	*23*	*23*
Patronised	*24*	*24*
Challenged	*25*	*25*
Comfortable	*26*	*26*
Happy	*27*	*27*
Warm	*28*	*28*
Stressed	*29*	*29*
Disbelief	*30*	*30*
Worried	*31*	*31*

Q2. Thinking about how living with hypertension makes you feel, please indicate to what extent you agree or disagree with the following statements.
SHOWCARD + ROTATE ORDER OF STATEMENTS

		Strongly Disagree						Strongly Agree
**Single answer per row**		**1**	**2**	**3**	**4**	**5**	**6**	**7**
**1**	I feel as though I am ill	**1**	**2**	**3**	**4**	**5**	**6**	**7**
**2**	I am constantly reminded of the fact that I have hypertension	**1**	**2**	**3**	**4**	**5**	**6**	**7**
**3**	Having hypertension means I cannot lead a normal life	**1**	**2**	**3**	**4**	**5**	**6**	**7**
**4**	I find myself wondering "Why me?!"	**1**	**2**	**3**	**4**	**5**	**6**	**7**
**5**	I feel different from all my friends and colleagues	**1**	**2**	**3**	**4**	**5**	**6**	**7**
**6**	I have accepted that hypertension is part of who I am	**1**	**2**	**3**	**4**	**5**	**6**	**7**
**7**	People forget that I have different needs	**1**	**2**	**3**	**4**	**5**	**6**	**7**
**8**	People who don't have hypertension don't understand what it is like for me	**1**	**2**	**3**	**4**	**5**	**6**	**7**

Q3. (a) Please now indicate the extent to which you agree or disagree with the following statements. These statements deal with how you feel your friends and family may perceive and treat your condition.
SHOWCARD + ROTATE ORDER OF STATEMENTS

		Strongly Disagree						Strongly Agree
**Single answer per row**		**1**	**2**	**3**	**4**	**5**	**6**	**7**
**1**	My friends and family are supportive	**1**	**2**	**3**	**4**	**5**	**6**	**7**
**2**	My friends and family are aware of my condition and the implications	**1**	**2**	**3**	**4**	**5**	**6**	**7**
**3**	My friends and family encourage me to control my hypertension	**1**	**2**	**3**	**4**	**5**	**6**	**7**
**4**	My friends and family seem more concerned about my hypertension than I am	**1**	**2**	**3**	**4**	**5**	**6**	**7**
**5**	My friends and family don't care about my condition as much as I do	**1**	**2**	**3**	**4**	**5**	**6**	**7**
**6**	My friends and family 'advise' me about what I should do differently	**1**	**2**	**3**	**4**	**5**	**6**	**7**

Q3. (b) Please now indicate the extent to which you agree or disagree with the following statements. These statements deal with your relationship with the doctor who treats your hypertension.
SHOWCARD + ROTATE ORDER OF STATEMENTS

		Strongly Disagree						Strongly Agree
**Single answer per row**		**1**	**2**	**3**	**4**	**5**	**6**	**7**
**1**	My doctor takes my hypertension seriously	**1**	**2**	**3**	**4**	**5**	**6**	**7**
**2**	My doctor listens to my concerns about hypertension and provides helpful answers	**1**	**2**	**3**	**4**	**5**	**6**	**7**
**3**	My doctor is sometimes annoyed that I am not managing my hypertension very well	**1**	**2**	**3**	**4**	**5**	**6**	**7**

**4**	My doctor is unwilling to spend more time discussing with me about my condition	**1**	**2**	**3**	**4**	**5**	**6**	**7**
**5**	My doctor gives me the feeling that my hypertension is my fault	**1**	**2**	**3**	**4**	**5**	**6**	**7**
**6**	My doctor makes unrealistic suggestions about the way I should live	**1**	**2**	**3**	**4**	**5**	**6**	**7**

Q4. To what extent has hypertension impacted your life in terms of the following?
(Please use 7 scales, 1= No impact at all, 7 = Significant impact) **(Rotate order of statements)**
SHOWCARD + ROTATE ORDER OF STATEMENTS

**Single answer per row**		**No impact at all**						**Significant impact**
1	Ability to work	**1**	**2**	**3**	**4**	**5**	**6**	**7**
2	Ability to engage in leisure activities	**1**	**2**	**3**	**4**	**5**	**6**	**7**
3	Sexual drive / desire to have sex	**1**	**2**	**3**	**4**	**5**	**6**	**7**
4	Ability to drive a car / motor vehicle	**1**	**2**	**3**	**4**	**5**	**6**	**7**
5	Ability to sleep	**1**	**2**	**3**	**4**	**5**	**6**	**7**
6	My general mood	**1**	**2**	**3**	**4**	**5**	**6**	**7**
7	Ability to engage in family activities	**1**	**2**	**3**	**4**	**5**	**6**	**7**
8	Ability to have sex	**1**	**2**	**3**	**4**	**5**	**6**	**7**
9	Ability to plan for the future	**1**	**2**	**3**	**4**	**5**	**6**	**7**
10	Overall impact on my Quality of life	**1**	**2**	**3**	**4**	**5**	**6**	**7**

Q5. Which one of the following statements most closely reflects your opinion on publicity surrounding hypertension (e.g. in advertisements, public information and the news?)

	SC
I feel there should be more publicity to raise awareness of hypertension and what health risks it can cause	***1***
I think the current level of publicity given to hypertension is about right	***2***
I feel there is too much publicity about hypertension; too many constant reminders	***3***

Q6. Please rank the following factors in order of importance for motivating you to keep your hypertension under control? **Rank the most important factor as "1", the next as "2", and so on.** **(Rotate order of statements)**
SHOWCARD + ROTATE ORDER OF STATEMENTS

Force unique ranking

	**Factors**	**Ranking**
**1**	Avoiding future health problems linked to hypertension	
**2**	Avoiding having to increase dosage of medication	
**3**	Avoiding 'high' blood pressure levels	
**4**	Avoiding 'low' blood pressure levels (hypotension)	
**5**	Minimising the impact on my everyday life	
**6**	Minimising the worry for my family and friends	
**7**	Reducing the no. of follow-up visits with my doctor	
**8**	Knowing that my family still needs me	
**9**	Knowing that one day I can stop taking medication	
**10**	Keeping my blood pressure level at my target levels	

Q7. Besides taking your prescription medication for hypertension, which of the following do you do to manage your hypertension?
Record spontaneous answers first, then prompt.
ROTATE ORDER OF STATEMENTS when prompting

	MC
Use homeopathic remedies	***1***
Manage my stress levels e.g. take time off work	***2***
Herbal supplements available at retail pharmacies	***3***
Cut out/reduce certain food from my diet	***4***
Others specify:	

BP Goal & Measurement

Q8. How frequent is your blood pressure being measured?

	SC
Once every 6 months	***1***
Once every 3 months	***2***
Once every month	***3***
Once every 2 weeks	***4***
Once a week	***5***
1-2 times **per week**	***6***
3-4 times **per week**	***7***
5-6 times **per week**	***8***
Once **per day**	***9***
Twice **per day**	***10***
Three times **per day**	***11***
More than three times **per day**	***12***
Never	***13***
**(DO NOT READ OUT)** I can't remember	***99***

Q9. Usually, who measures your blood pressure levels?

	SC
Self	***1***
Doctor/ nurse	***2***
Caregiver (e.g. spouse/ relative/ friend)	***3***
Other: Please specify ____________________	

Q10. Do you currently own a blood pressure monitor?

	SC	Routing
Yes	**1**	**Proceed to Q11**
No	**2**	**Skip to Q16**

Q11. How long have you had this blood pressure monitor?

		Years			months

Logic check: Q11 ≤ S4

Q12. How often do you use the blood pressure monitor?

	SC
Once every 6 months	*1*
Once every 3 months	*2*
Once every month	*3*
Once every 2 weeks	*4*
Once a week	*5*
1-2 times **per week**	*6*
3-4 times **per week**	*7*
5-6 times **per week**	*8*
Once **per day**	*9*
Twice **per day**	*10*
Three times **per day**	*11*
More than three times **per day**	*12*
Never	*13*
I can't remember	*99*

Q13. Compared with when you first bought your own blood pressure monitor till now, do you:
SHOWCARD

	SC
Test my BP **much more frequently** now compared to before	*1*
Test my BP **a little more frequently** now compared to before	*2*
Test my BP **as frequently** now compared to before	*3*
Test my BP **a little less frequently** now compared to before	*4*
Test my BP **much less frequently** now compared to before	*5*
**Do not test** my blood pressure levels anymore	*6*

Q14. (Deleted)

Q15. Who recommended the blood pressure monitor to you?

	MC
Doctor recommendation	**1**
Pharmacist recommendation	**2**
Other patient's recommendation	**3**
Internet search	**4**
Recommendation from hypertension association	**5**
Other (please specify)	

ASK Q16 IF Q10/CODE 2 (Do not own BP Monitor)

Q16. Do you **want to have** a blood pressure monitor in your home?

	SC
Yes	**1**
No	**2**

ASK Q17 IF:
Q10/CODE 2 (Do not own BP Monitor)OR
Q13/CODE 6 (Do not use BP Monitor anymore)

Q17. Which of the following is the most important reason why you don't use a blood pressure monitor?
DO NOT READ OUT

	SC
I don't know what a blood pressure monitor is	***1***
I believe blood pressure monitoring should be performed by doctors	***2***
I have been told that it is not necessary for me	***3***
I find I can get by without using one	***4***
I don't like the idea / don't want to use one	***5***
I don't like the process itself	***6***
The cost was too high	***7***
I used one but it was too time-consuming	***8***
I used one but I kept forgetting	***9***
Others, please specify:_________________	

ASK ALL
Q17B. Does your doctor monitor your blood pressure measurements? (Checking/ monitoring Patient BP levels over a period of time)

	SC
Yes	**1**
No	**2**

Q18. (a) What is your personal blood pressure target goal?
(b) At what blood pressure level will you be concerned about your condition?

**(A)**	**Reading**	**Reference (DO NOT READ OUT)**
Systolic	mmHG	**Range: 90~230 mmHG**
Diastolic	mmHG	**Range: 60~140 mmHG**

**(B)**	**Reading**	**Reference (DO NOT READ OUT)**
Systolic	mmHG	**Range: 90~230 mmHG**
Diastolic	mmHG	**Range: 60~140 mmHG**

Logic check: (B) Should be equal to or higher than (A)

Q19. Please share with me what do you think happens when you blood pressure is above this target goal mentioned **in Q18a**? Please be as detailed as possible.

Q20. Which of the following statements best describes your feelings when you do not reach your blood pressure target goal in **Q18a**?
SHOWCARD + ROTATE ORDER OF STATEMENTS

	SC
I am extremely concerned about reaching my target blood pressure level and will be very upset if I do not reach it consistently	***1***
I believe that I have done my best to reach my target blood pressure level, so it is fine even if I do not reach my target levels.	***2***
I am already on medication so I'm not overly concerned about reaching my target blood pressure levels.	***3***
Others specify:	

Consultations with Doctor & Compliance

Q21. Which doctor type listed here are you currently seeing to manage your hypertension?

	SC
GP (General Practitioner)	*1*
Cardiologist	*2*
Nephrologist	*3*
Others, please specify:________________	

Q22. (a) How often did you see your doctor when you were first diagnosed with hypertension?
(b) How often do you see you doctor now?

	(a) First Diagnosed	*(b) Current*
Once every 6 months	*1*	*1*
Once every 3 months	*2*	*2*
Once every month	*3*	*3*
Once every 2 weeks	*4*	*4*
Once a week	*5*	*5*
When I need to/ feel like it	*6*	*6*
Others specify:		

Q23. Does anyone from your family or friends/ relatives accompany you on your doctor visits?

	**SC**
Yes	**1**
No	**2**

Q24. Thinking of the current medication that you are currenly taking, how often do you take your hypertension medication?
SHOWCARD

	SC	*Routing*
Once every 2 weeks	*1*	**Proceed to Q25A then Q26**
Once a week	*2*	
1-2 times **per week**	*3*	
3-4 times **per week**	*4*	
5-6 times **per week**	*5*	
Once **per day**	*6*	
Twice **per day**	*7*	**Proceed to Q25Bi then Q26**
Three times **per day**	*8*	
More than three times **per day**	*9*	
When I can remember to take it	*10*	**Proceed to Q25A then**

		Q26
Others: Please specify		

ASK Q25A IF patients take it once a day or less (Q24/CODE1-6, 10)

Q25. (A) At what time of the day do you take your hypertension medication?

	SC
Morning	***1***
Afternoon	***2***
Evening	***3***
When I can remember to take it	***4***

ASK Q25Bi and Q25Bii IF patients take it more than once a day (Q24/CODE 7-9)

Q25 (B)(i) At what time of the day do you first take your hypertension medication?
Single Answer
(B)(ii) Subsequently, when do you take your medication?
Multiple Answers

	Bi: First take medication	***Bii: Subsequent***
Morning	***1***	***1***
Afternoon	***2***	***2***
Evening	***3***	***3***
When I can remember to take it	***4***	***4***

ASK ALL

Q26. Who gave you these instructions on taking your medication?

	SC
Doctor (GP/Cardiologist/Nephrologist/Other doctors)	***1***
Retail Pharmacist	***2***
Family/ Friends	***3***
I came up with my own schedule	***4***
Others, please specify:_________________	

Q27. Some people find it difficult to follow their doctor's instructions when taking prescribed medication, sometimes forgetting to take the occasional dose. How closely do you follow your doctor's instructions for your hypertension treatment, please use a scale of 1 to 7, where 1 means you rarely follow your doctor's instructions and 7 means you always follow your doctor's instructions.

Single answer

I rarely follow my Doctor's instructions						***I always follow my Doctor's instructions***
1	2	3	4	5	6	7

Q28. Thinking about when getting a new prescription for your hypertension medication, which of the following best describes your typical behaviour. Please circle one option only.

	**SC**
I always get a new prescription before my old one runs out so I am never without my hypertension medication	**1**
I usually get a new prescription before my old one runs out but sometimes I do not have my hypertension medication for a day or two.	**2**
There is usually a gap of one or two days between when my old prescription runs out and getting a new prescription for hypertension medication	**3**
There is usually a gap of more than two days between when my old prescription runs out and getting a new prescription for hypertension medication	**4**
Other answer, please specify	**5**

Q29. (a) Which of the following specific pieces of advice have been given to you by healthcare professionals (E.g. Doctors/ nurses) in connection with your hypertension within the last year? (Interviewer: Rotate order of answer options, and tick all that apply)
Multi-code
(b) Thinking about all these suggested lifestyle changes; to what extent did you act on this suggestion? Please use 7 scales: 1=Not followed at all, 7= Follow their suggestions exactly. **(Ask Q29b only for statements selected in Q29a)**
Single code per row
SHOWCARD + ROTATE ORDER OF STATEMENTS

LOGIC CHECK: IF CODE 14 IS SELECTED, NO OTHER CODES SHOULD BE SELECTED & SKIP (B)

	**Specific pieces of advice**	**(A) Ever been given by healthcare professionals**	**(B) Action on suggestion 1=Not followed at all, 7= Follow their suggestions exactly**						
**1**	Eat less in general	**1**	1	2	3	4	5	6	7
**2**	Eat more healthily	**2**	1	2	3	4	5	6	7
**3**	Use “calorie counting” as part of diet	**3**	1	2	3	4	5	6	7
**4**	Cut out certain foods	**4**	1	2	3	4	5	6	7
**5**	Reduce salt intake	**5**	1	2	3	4	5	6	7
**6**	Exercise more in general	**6**	1	2	3	4	5	6	7
**7**	Exercise to a specific routine (e.g. 30 minutes, 3 times per week)	**7**	1	2	3	4	5	6	7
**8**	Smoke less	**8**	1	2	3	4	5	6	7
**9**	Stop smoking	**9**	1	2	3	4	5	6	7
**10**	Drink less alcohol	**10**	1	2	3	4	5	6	7

**11**	Stop drinking	**11**	1 2 3 4 5 6 7
**12**	Take vitamins / supplements routinely	**12**	1 2 3 4 5 6 7
**13**	Start using a blood pressure monitor	**13**	1 2 3 4 5 6 7
**14**	None of these	**14**	

Q30. After being diagnosed by a healthcare professional please tell me what are the sources of information you have accessed to understand more about hypertension and how to manage it?

	MC
Doctor	***1***
Family/ relatives	***2***
Drug store/retail pharmacist	***3***
Friends	***4***
TV	***5***
Internet chat forums	***6***
Print materials like newspaper and magazines	***7***
Conferences and talks	***8***
Internet/ search websites	***9***
Others, please specify _______________	
Did not attempt to access any source of information	***98***

Attitudes towards General Health & Lifestyle

Q31. Please look at the following statements that talk about different attitudes to health and wellbeing and indicate your level of agreement / disagreement with each.
SHOWCARD + ROTATE ORDER OF STATEMENTS

		**Strongly Disagree**						**Strongly Agree**
**Single code per row**		**1**	**2**	**3**	**4**	**5**	**6**	**7**
**1**	I spend a lot of time thinking about my health	**1**	**2**	**3**	**4**	**5**	**6**	**7**
**2**	A lot of what I do centres around being / staying healthy	**1**	**2**	**3**	**4**	**5**	**6**	**7**
**3**	I only think about my health when I am feeling ill	**1**	**2**	**3**	**4**	**5**	**6**	**7**
**4**	I am very focused on eating healthily	**1**	**2**	**3**	**4**	**5**	**6**	**7**
**5**	I am careful not to consume too much alcohol	**1**	**2**	**3**	**4**	**5**	**6**	**7**
**6**	I always find / make time to ensure that I eat properly	**1**	**2**	**3**	**4**	**5**	**6**	**7**
**7**	I am careful on lowering my salt intake	**1**	**2**	**3**	**4**	**5**	**6**	**7**
**8**	I don’t monitor what I eat usually	**1**	**2**	**3**	**4**	**5**	**6**	**7**
**9**	Physical exercise plays an important role in my life	**1**	**2**	**3**	**4**	**5**	**6**	**7**

**10**	I am often too tired to exercise	**1**	**2**	**3**	**4**	**5**	**6**	**7**
**11**	I find exercising to be a chore	**1**	**2**	**3**	**4**	**5**	**6**	**7**
**12**	I can always find time to exercise	**1**	**2**	**3**	**4**	**5**	**6**	**7**
**13**	I believe that keeping my stress level low is more important than diet and exercise	**1**	**2**	**3**	**4**	**5**	**6**	**7**
**14**	A highly stressed person is an unhealthy person	**1**	**2**	**3**	**4**	**5**	**6**	**7**
**15**	Stress is something I cope very well with	**1**	**2**	**3**	**4**	**5**	**6**	**7**
**16**	I believe that stress causes my hypertension condition to worsen	**1**	**2**	**3**	**4**	**5**	**6**	**7**

Q32. Since being diagnosed with hypertension till now,
(a) How has your exercise regime changed? Please select one option from the list below.
SHOWCARD

	SC
Doing **significantly less** exercise	***1***
Doing **slightly less** exercise	***2***
**No change** from before diagnosis of hypertension	***3***
Doing **slightly more** exercise now	***4***
Doing **significantly more** exercise now	***5***

(b) How has your diet changed? Please select one option from the list below.

SHOWCARD

	SC
My dietary habits have become **significantly less** controlled	***1***
My dietary habits have become **slightly less** controlled	***2***
**No change** from before diagnosis of hypertension	***3***
My dietary habits have become **slightly more** controlled	***4***
My dietary habits have become **significantly more** controlled	***5***

(c) How has your attitude towards stress management changed? Please select one option from the list below.

SHOWCARD

	SC
My stress levels have become **significantly less** controlled	***1***
My stress levels have become **slightly less** controlled	***2***
**No change** from before diagnosis of hypertension	***3***
My stress levels have become **slightly more** controlled	***4***
My stress levels have become **significantly more** controlled	***5***

Q33. In a typical week, on how many days will you engage in at least 30 minutes of aerobic exercise (e.g. cycling, walking, running, sports, and gym)?
Logic Check: Range = 0- 7
Don't Know = code 99

☐☐ Day (s) per week

Q34. (a) Which of the following conditions are you aware of, that you know can be related to hypertension? (Multiple answers allowed)
(b) And which of the following conditions are you aware of, that can be caused by uncontrolled blood pressure levels? (Multiple answers allowed)
(c) Please indicate how worried you are about each of these potential health problems. Please use 7 scales, 1=Not at all worried, 7=very worried (those selected in (a))

**Conditions**	**(a)**	**(b)**	**(c) How worried about each condition**						
			Not at all worried						Very worried
Renal (kidney) problems	**1**	**1**	1	2	3	4	5	6	7
Heart attack/ failure	**2**	**2**	1	2	3	4	5	6	7
Problems with vision / eyes	**3**	**3**	1	2	3	4	5	6	7
Problems with circulation / feet, hands (e.g. ulcers)	**4**	**4**	1	2	3	4	5	6	7
Nerve problems (loss of feeling, numbness, neuropathic pain)	**5**	**5**	1	2	3	4	5	6	7
Stroke	**6**	**6**	1	2	3	4	5	6	7
Coronary artery disease	**7**	**7**	1	2	3	4	5	6	7
Respiratory problems	**8**	**8**	1	2	3	4	5	6	7
Dementia	**9**	**9**	1	2	3	4	5	6	7
Eczema	**10**	**10**	1	2	3	4	5	6	7
Other, please specify:___________________									

Q35. Please tell us your height in centimetres.

			cm

Logic Check: Height to be between 130cm – 220cm

Q36. Please now tell us your weight in kilograms.

			Kg

Logic check: Kg range: 32kg~200kg

Q37. What is your waist measurement?

			inches

or

			cm

Logic Check:
- ***inches range: 18~48 inches***
- ***cm range: 45cm~122cm***

Don't know – code 999

Thank and close

1348 Edvantage Patient Focus Group Research
Discussion Guide

1. Introduction (5 min)

Objective: Introduction & warm up

↓

2. Understanding Hypertension (30 min)

Objective: Gain insights + understanding to the perceptions & attitudes towards managing their Hypertension

↓

3. Pre- and Post-diagnosis of Hypertension (20 min)

Objective: Extent to which patients lifestyle, attitude and level of concern has altered since living with this condition

↓

4. Consultation with doctor (interactive group exercise) (30 min)

Objective: Identify gaps between patients' expectations and actual experience & information received at the clinic

↓

5. Patient Profiles testing (30 min)

Objective: Using patient quotes to elicit further understanding on 1) Monitoring of BP levels – how important is it to them? 2) BP being uncontrolled – what does this mean to patients 3) Myth on hypertension 4) Ways of managing their hypertension 5) Side effects concerns

↓

6. Warm down (5 min)

Total: 120 minutes

Section 1 – Introduction (5 min)

- Thanks respondents for attending the FGD
- Explain market research, introduce ”The Research Partnership”, self + note taker
- No right or wrong answers, everyone’s opinion is important and should be honestly expressed, free to agree and disagree with each other
- Explain audio / video recording and viewing
- Reassure confidentiality
- Explain objectives of the study
- Get respondents to introduce self :
    - Hobbies, length of time living with hypertension, any upcoming plans they have in the coming year
    - Explore co-morbidities including treatment. How long have they suffered from each co-morbidity?

Section 2 – Understanding Hypertension (20 minutes)

Objective:
Gain insights + understanding to the perceptions & attitudes towards managing their Hypertension

Explain that today we are going to discuss something that all respondents have in common – hypertension

Understanding hypertension

Prior to attending tonight’s focus group you all have completed a life world journal where you explained what it is like to live with hypertension.

Can everyone please have their life world journal in front of them

Moderator: select one person to read out their answer to question 1:

1. **LWJ – Q1** Imagine a friend who has just been diagnosed with hypertension…how would you answer her.. (Symptoms, danger, treatment, how to know if you're doing well etc.)

- Can anyone else relate to this? Did anyone else record something similar?
- Did anyone else record something differently?

Moderator: select a different person to read out their answer to LWJ question 1.

Repeat above until all comments are read out

- Where do you get/find information for your hypertension? Probe: Doctor, family, friends, other patients with hypertension?
- How do you feel about the level of public awareness on hypertension in <insert country>? Probe: More or less publicity? Just enough? Why?
- What information relating to hypertension would you like to receive in the future? Which source of information do you prefer to deliver such information to you? Probe: Doctor, family, friends, hospitals, patient support group
  - Why?

Moderator: select one person to read out their answer to LWJ question 2:

2. **LWJ – Q2** How do you feel about having this condition? When I think about my hypertension, I FEEL…(DESCRIBE IN DETAIL HOW YOU FEEL PHYSICALLY, EMOTIONALLY, PSYCHOLOGICALLY)

3. I would like to understand how you were first diagnosed with HTN. **Who knew/suspected** they were suffering from HTN before they were diagnosed?
   - And who **had no idea** they had HTN before they were diagnosed?

MODERATOR SELECT THE GROUP WHO KNEW/SUSPECTED THEY HAD HTN

4. What made you think you had HTN?
   - How long did you suspect you were suffering from HTN before you went to a doctor?

- What symptoms did you experience if any?
- Did you take any self-medication (non-prescription) to ease these symptoms before seeing a doctor?
- If yes: Why? What forms of self-treatment did you do? How did you find out about these methods?
- Do you still use these methods to ease your symptoms? Why so?

5. Can anyone else relate to this? Did anyone else record something similar?
6. Did anyone else record something differently?

MODERATOR ASK THOSE WHO DID NOT KNOW THEY HAD HTN

7. When you were first diagnosed:
   - How concerned were you when you were first diagnosed? Why do you think you felt that way?
   - How much did you know about HTN prior to being diagnosed?
8. Can anyone else relate to this? Did anyone feel this way?
   - Did anyone else feel differently?

MODERATOR ASK ALL

When you were first diagnosed who went out and got a BP (blood pressure) meter to measure their BP levels at home/work?

- **IF RESPONDENT BOUGHT A BP METER ASK:**
  - Why did you get a BP meter? How often did you check your BP levels?
  - How often did you see your doctor?
  - How often do? At the doctor or at home?
  - Compared to the other conditions that you might have – how serious do you perceive your hypertension to be relative to these other conditions? Why do you say so?

9. **IF RESPONDENT DO NOT HAVE A BP METER ASK:**
   - Do you want a BP meter in your home? Why/ Why not?
   - Do you think it is necessary for you to have one? What are your concerns on getting a BP meter? Probe: Costs factors, importance of having a BP meter to control your blood pressure levels
10. I would now like to understand your perspective on blood pressure (BP) control:

- What is your current BP level? **MODERATOR WRITE ON FLIP CHART**
- What do you consider to be your target BP goal? **MODERATOR WRITE ON FLIP CHART – GAIN CONSENSUS ON WHICH IS THE MOST COMMON TARGET BP GOAL**
- At what point of your BP level will you begin to feel worried/ concerned? Why do you say so? **MODERATOR WRITE ON FLIP CHART – GAIN CONSENSUS ON WHICH IS THE MOST COMMON LEVEL THEY WORRY ON**
- I understand that BP levels will fluctuate throughout the day. How do you feel when your BP levels is higher than the mentioned target BP goals? Why do you say so?
- Did your doctor ever explain why it is important to keep your BP under control? Why/ why not? **Probe:** Other conditions hypertension might cause
    - **YES:** What did he/ she say about uncontrolled BP levels?

Section 3 – Pre- and Post-diagnosis of Hypertension (20 minutes)

Objective:
Extent to which patients lifestyle, attitude and level of concern has altered since living with this condition

11. All of you have lived with hypertension for some time now. So I would like to understand:
    - How, if at all, has your opinion towards hypertension changed since you were first diagnosed?
    - Compared with when you were first diagnosed how do you feel now?
    - Has this condition affected you and your family members? In what ways? How do you feel about this?
    - After being diagnosed with hypertension, has your lifestyle change compared to before you had this condition? Probe:
        - Hobbies? Future plans? In what ways?
        - How does this make you feel?
    - Compared with before are you checking your BP level less, the same or more than when you were first diagnosed?
        - Explore reasons behind the change in BP measurement.

MODERATOR WRITE ON FLIP CHART UNDER 2 COLUMN HEADINGS:
NOTE (DO NO READ OUT): THE AIM OF THIS SERIES OF CHANGES IS TO UNDERSTAND WHAT DRIVES THESE BEHAVIOURS

FIRST DIAGNOSED	NOW

12. I would like to understand how, if at all, your attitude and/or behaviour towards hypertension have changed between when first diagnosed and now.
    MODERATOR EXPLORE ALL CHANGES AND THEN ASK WHY. TAKING EACH CHANGE & ASK:

- o Why has your attitude/behaviour changed? Why do you act/ react differently now?

13. So overall to what extent do you feel that **you are in control of the treatment** of your hypertension? Why?

    Would you like to take greater control of your condition?

    IF YES ASK:

    - ▪ Why?
    - ▪ How would you judge/evaluate if you are in control?
    - ▪ How would this work on a practical/real-life basis?

    - o Why do you need to stay in control?
    - o What will happen if you do not stay in control? Why do you think so?
    - o What motivates you to stay in control? Why do you say so?

Now's lets go back to your **LWJ that you complete and refer to Q6**

14. **LWJ – Q6** What frustrates you most about any aspect of your hypertension. Sources of frustration can be related to medication, the impact hypertension has on the way you look at life or anything at all. Please read out some of the things you have written here to describe any frustrations that you feel.

    MODERATOR - HAVE EACH RESPONDENT READ OUT A FRUSTRATION

    ON A FLIP CHART GROUP THE FRUSTRATIONS UNDER COMMON THEMES (EG DIET & EXERCISE, TREATMENT, DOC CONSULTATION ETC)

    PROBE: If patients mention taking a lot of pills, do they mean the frequency per day or the no. of pills each time?

15. Have the group to vote/prioritise frustrations to understand what has the biggest impact on them as hypertension patients living in (insert country)

16. Conversely – what encourages them to continue managing their hypertension? Probe: family/ friends, doctor support, plan for the future, hope of.

Section 4 – Consultation with doctor (30 minutes)

Objective:
Identify gaps between patients' expectations and actual experience & information received at the clinic

Moderator: I would like you to now imagine I am the usual doctor you visit for your hypertension condition (interactive exercise):

17. Please share with me in as much detail as possible, what is usually discussed during the visit?
    - Do you provide the doctors with any information? Probe: BP reading, other medications for other conditions?
    - Will you usually ask the doctor (me) any questions?
    - What questions would you usually ask the doctor (me)?
    - What would I say (as a doctor) in response to the questions you just asked?
    - Are there any areas you won't discuss with the doctor on? Why?
    - Did the doctor explain to you about the usage and side effects of your medication? Why/ why not?
        - YES: What did he/she say?
        - NO: Do you want your doctor to share with you the usage and side effects of your hypertension medication? Why/why not?
        - Anything else you would like him/her to explain on the medication?

16. **Now please imagine I am the most respected hypertension doctor in the city and you are in a seminar together with the other patients. You have as long as you want to ask me as many questions as you like.**
    a. Would you ask me any questions? Why/ Why not?
    b. IF YES: What questions would you ask me? Please be as detailed as possible.

17. **Imagine now you can only ask me one question – what one question would you ask me?**

- Why would you ask this particular question and nothing else?
- Overall, how would you describe your relationship with your doctor? Are you comfortable with the level of conversation & interaction you have with him/her on hypertension? Why do you say so?
  - Is there anything he/she can do better to help you in managing your hypertension? Probe: information on medication, disease, support. Please be as specific as possible.

18. **REFER TO LWJ Q5** - Please draw a line on this chart to represent how you feel when your doctor tells you your blood pressure is **above** your target level.
    - Please describe why you feel this way when your blood pressure is above target? Please consider the following points
      - What are you most concerned about?
      - What does your doctor think when your BP is above target?
      - What does your family/friends think when your BP is above target?

    MODERATOR ENSURE ALL RESPONDENTS HAVE READ OUT THEIR CONCERNS

19. How many of you were **previously on a different brand of medication** for your hypertension?
    - If yes:
      - Can you recall what that medication was?
      - Why did you switch to your medication? Probe: Was it your decision or your doctor's decision or both?
      - Did the doctor explain to you the reasons for this switch? How did they explain it? Probe: side effects, effectiveness, frequency of taking the medication
      - What further information would you have liked to discuss or for your doctor to explain on this switch?
      - What questions did you have when you left the doctor's office?

- How did you feel about the switching of your medication? Concerned/reassured? Why do you say so?

20. Imagine on your next visit to your doctor they say that **they would like to switch your medication to a new product**:
    - How would you feel if your Dr suggested to switch your medication?
    - What information do you want your doctor to give you?
    - What questions do you want to ask?
    - How frequently do you want to follow up with your Dr?
    - How would you evaluate whether the new brand is working?

Section 5 – Patient Profiles Stimulus (30 minutes)

Objective:
Using patient quotes to elicit further understanding on 1) Monitoring of BP levels – how important is it to them? 2) BP being uncontrolled – what does this mean to patients 3) Long term concern on taking hypertensive medication 4) Ways of managing their hypertension 5) Side effects concerns

MODERATOR READ OUT:

I am going to show you a few slides on what hypertension patients say on their condition and I would like to get your reaction to this. Remember there are no right or wrong answers it is your personal opinion I am interested in:

21. **Patient Profile 1 (Monitoring BP levels)**
    - *"When I first bought the blood pressure meter I checked like 5-7 times a day, now… I don't check that often. I usually check it before I see a doctor just to be safe"*
        - Probe:
            - To what extent can you relate to his/her behaviour? Why?
            - Why do you think this person initially checked 5-7 times a day?
            - Will you express these thoughts to the doctor? Have you done so?
            - Why do you think this person checked less frequently now?
            - What does he mean by 'just to be safe'?
22. **Patient Profile 2 (Controlling BP)**
    - *"My blood pressure level is usually ok, I think around 140/90. I don't feel anything so I should be fine."*
        - Probe:
            - To what extent can you relate to his/her behaviour? Why?

- Do you agree that 140/90 is ok?
- Will you express these thoughts to the doctor? Have you done so?
- Extent to which she should check her BP? When should she check it? Any symptoms she should look out for?

23. **Patient Profile 3 (Myth)**
    - o “I hope one day I can stop this high blood pressure medication.”
        - Probe:
            - To what extent can you relate to his/her behaviour? Why?
            - Is it possible to stop medication one day?
            - Will you express these concerns to doctor? Have you done so?

24. **Patient Profile 4 (Ways of managing their hypertension)**
    - o “When I first found out I had hypertension, I tried all sorts of things like changing my food, drinking bitter gourd juice, going for morning walks etc. Now I just take my medication every day, try to be careful on what food I eat, but other than that I can’t do much anymore”
        - Probe:
            - To what extent can you relate to his/her behaviour? Why?
            - Self-treatment – what do you do? Do you think it works?
            - Will you express these thoughts to the doctor? Have you done so?

25. **Patient Profile 5 (SIDE EFFECTS)**
    - o “I worry about the long-term effects of taking hypertension medication”
        - Probe:
            - To what extent can you relate to his/her behaviour? Why?

Commented [PL1]: Please change this to an equivalent in your market. For example, some patients in Hong Kong believe in medicinal herbs may help to alleviate their hypertension, please make this urban myth specific to your country.

- Will you express these concerns to doctor? Have you done so?

Section 6 – Warm Down (5 minutes)

- This is the final section and we will be finished in a short time but before we go let me ask you this - I would like you to think about any aspect of your hypertension. What is the single most frustrating thing about your hypertension? **MODERATOR: WRITE EACH FRUSTRATION ON POST- IT NOTES**
  - PROBE: Why is this the single most frustrating aspect?
  - How can these frustrations be eliminated/reduced?

- Besides curing your condition what could be done to improve your quality of life? Probe:
  - o if that was improved how would it impact on your quality of life?

- To what extent do you feel that you are in control of the treatment of your hypertension? PROBE: Would you like to take greater control of your condition? IF YES ASK: How would this work on a practical basis?

- The objective of this discussion was to understand hypertension from the patient's point of view.

- What final comments/feedback would you like to give in summary?

THANK AND CLOSE

1348

Project Edvantage

Discussion Guide (GPs/Cardiologists)

**Introduction (2-3 minutes)**

General introduction:
Thank you for agreeing to take part in this interview. My name is **[insert name]** working on behalf of The Research Partnership, a global, independent medical market research agency. We will be tape-recording the interview, both audio and video, and there are some interested colleagues and clients viewing this interview. However, I would like to remind you that everything you say will be kept completely confidential. Please also note that there are no right or wrong answers, we are just interested in your thoughts and opinions.

Explain purpose of interview:
The purpose of this interview is to discuss your treatment and management of hypertension. I will also be showing you some data and information for a new hypertension drug about to be launched.

Please note that this is for market research purposes only – it is in no way intended to be promotional.

Section 1 – Hypertension Treatment Approach: Context Setting (7 mins)

Objective: Set the context for the rest of the interview through exploration of usage /perceptions of ARBs and FDCs, drug compliance and current unmet needs

1) Please briefly tell me a bit about the type of practice that you work in

   Probe:

   - Public / Private
   - NOT GP/IM: Office / Hospital

2) Approximately, how many patients do you see in a typical month?

   - Of these, what proportion suffers from hypertension?
     - o What proportion of these patients would you say have good compliance to their hypertension medication?
     - o What patients are likely to be less compliant?
       - ▪ PROBE:
         - Those with co-morbidities?
         - Elderly?
         - Those on lower incomes?
       - ▪ Compared with other chronic conditions how would you describe patient compliance with hypertension treatment, overall would you say its better, the same or worse?

3) What is the role of ARBs in the management of hypertension?

   - As a class, what are the main advantages and drawbacks of ARBs?
   - Which patients do you prescribe ARBs to?
   - When do you typically start patients on ARBs?

4) What brands of ARBs do you prescribe? **Record Answer on Self Completion Sheet 1**

   If multiple ARB brands used ask:

   - If we take your total ARB patients as 100%, approximately what is the approximate percentage of patients receiving each brand of ARB? **Record Answer on Self Completion Sheet 1**
   - How do you decide which ARB brands to use?
     - o Does your brand selection differ according to different hypertension patients? How? Please explain fully?

5) What dose of ARBs do you typically prescribe? **Record Answer on Self**

Completion Sheet 1

- How does this differ by
  - o Brand of ARB?
  - o Condition of the patient/patient type?

6) To, what extent, if at all, do you ever prescribe a half an ARB tablet?

   If yes: How frequently do you prescribe this?

   - Why do you do this?
   - How does this differ by ARB brand?
   - How does this differ by patient type?
   - What is the typical duration of treatment of this half tablet prescription?

   If no: Why do you think some physicians we have spoken prescribe half ARB tablets to their uncontrolled hypertension patients?

   When in the day, if at all, do you typically instruct the patients when to take their ARB?

   - o Morning, lunchtime, afternoon or evening, or no instructed time/when the patient prefers?
   - o Why this time?
   - o To what extent, if at all, does this differ by patient type?
   - o To what extent, if at all, does this differ by ARB brand?

7) What is the typical duration of treatment for ARBs? **Record Answer on Self Completion Sheet 1**

   - How does this differ by
     - o Brand of ARB?
     - o Condition of the patient/patient type?

8) To what extent, is you, choice of ARB brand dependent on patient's BP reading?

   - To what extent do you consider certain brands of ARBs to be for patients with a certain BP reading?
   - What brand? What BP?

9) To what extent is your ARB usage driven by treatment guidelines?

   - **If guideline driven:** What treatment guidelines?
   - To what extent, if at all, are these guidelines adhered to?

10) Do you prescribe fixed dose combinations (FDC) to your hypertension patients? Why/ why not?

If prescribe FDC ask: What FDCs do you prescribe? Please specify the molecules/brands.

- How frequently do you prescribe FDCs?
    - In what patient types?

11) What would you say are the main unmet needs in the management and treatment of hypertension?

- To what extent, if at all, are your hypertension patients failing to meet goal an unmet need? Please explain fully
- What proportion of patients would you estimate regular fail to meet the target Blood Pressure goals that are set for them?
    - How satisfied are you with this percentage?

- What proportion of patients who fail to achieve their BP goal would you describe as having good compliance with their hypertension medication?

Section 2 – Uncontrolled Hypertension: Context Setting (5 mins)

Objective: Understand physician perceptions of uncontrolled hypertension to provide context for the remaining interview

12) How do you define uncontrolled hypertension?

PROBE What do you base your definition on?**HAND RESPONDENT SHOWCARD 1:**

13) Please have a look at this table displaying different blood pressure ranges. Can you please indicate what proportion of your hypertension patient's fall into each of the ranges?
14) Out this ranges which would you classify as mild, moderate and severe hypertension?

15) What do you think are the reasons for your patients not reaching goal?

Probe:

- Patient lifestyle factors?
- Underlying disease/co-morbidities?

- Poor patient compliance to hypertension medication?
- Patient receiving inadequate/ineffective medication?
- Other reasons?

Of all the reasons you have given which do you consider are the main ones that prevent patients from achieving their goal?

16) Overall how concerned, if at all, are you when your hypertension patients fail to reach goal? Why do you say so?

When your patients are newly diagnosed with hypertension, how frequently do you see them for a consultation?

To what extent does this differ by patient type?

How do you know, when your hypertension has failed their first line of treatment?

- How is this monitored?
- How do you define treatment failure?
- What is your next step?
    - Do you switch/add on treatment? Which treatments? Why?
    - To what extent do you continue to monitor the patients instead of switch ort adding therapy?
        - How long do you monitor the patient for?
        - How many times would you see the patient before considering changing the treatment approach?

Section 3 – Uncontrolled patient profile and the Doctor/ Patient interaction (25 mins)

Objective: Detailed profiling of uncontrolled hypertension patient types and exploration of patient/doctor interaction, as well as rational/emotional drivers to disease management

For the next part of the interview, I would like to explore your uncontrolled hypertension patients in more detail.

17) Can you please explain to me some of the characteristics of your uncontrolled patients?

Probe:

- Co-morbidities
- Healthy lifestyle/non healthy lifestyle
- Age, Gender, Occupation
- Education level

18) To what extent do you recognize distinct different types of patients within the uncontrolled patient population you manage?
    - What are the different uncontrolled patient types? PROBE Fully

- For the next exercise I would like you to record for me the key characteristics of these different patient types so that I can have a detailed patient profile from you.

Hand Self Completion Sheet 2

Please refer to this self completion sheet. As you can see there are three patient profiles labeled A, B and C. Please complete a separate profile for three different uncontrolled patients that you have recently treated that represent typical patient types.

Please can you ensure they are distinct and different patients from each other and represent the different patient types we just discussed.

If you feel you have more patient types please let me know and I can provide extra profiles. On the other hand, if you feel you only have 1 or 2 patient types then you may complete less profiles.

Please note, the patient profiles contain demographics information as well as more clinical information such as co morbidities and current hypertension medication. There are also blank cells for you to add anything else that you think of interest about this patient

I would now like to discuss these patients in more detail.

Starting with Patient Profile A, ask:

- Can you please introduce this patient to me?
- Approximately what proportion of your uncontrolled hypertension patients does this patient represent?
- What is your current treatment approach for this patient? **Moderator: Ensure to get a clear understanding if mono therapy or combination and fixed dose combination versus free dose combination**
    - Why is this your treatment approach?
- How frequently do you see this patient for a consultation?

- When do you think, if at all, you will make a treatment adjustment for this patient?
    - What do you think this treatment adjustment will be?
        - **Probe**: Dose adjustment? A complete switch? Add on to existing treatment? Why?
        - What is your long term treatment and management plan for this patient?
- How compliant is this patient to their medication?
- **If patient is ever non compliant ask:**
    - How frequently does this patient skip/decide not to take their treatment?
    - Why do you think this patient is non compliant?
    - To what extent, does this patient, decide not to take their medication for extended periods/have a treatment holiday?
        - How long do they decide not to take their treatment for?
        - Why does the patient do this?
        - To what extent does the patient self medicate during this period?
- If patients has at least one co-morbidity ask:
    - To what extent, to see this patients uncontrolled hypertension as more , less or equaling concerning versus the other co-morbidities? Why?
        - What about the patient, are they more, less or equally concerned about the hypertension versus the other co-morbidities? Which co-morbidities? Why?
        - How does this impact compliance to their medication? Why?

Repeat above probes for the other patient profiles

I would now like to explore your interaction with your patients in more detail.

Hand Respondent Self Completion 3

For this exercise, please start with the patient profile which represents that largest uncontrolled patient group.

I would like you to complete this exercise by recording the interactions between yourself and the patient during the last consultation you had.

First of all I would like you to focus on the doctor's speech bubble. Please record what you said to this patient regarding not meeting their goal. If you discussed any next steps to their hypertension management/treatment adjustments, please record what you said and how you said it.

I would then like you to refer to the Doctor's thought bubble. Please record the thoughts and feelings you had when this patient was in front of you. Please remember these are the thoughts that you did not express to this patient and kept only to yourself.

Lastly please complete the patient thought/speech bubble. Please include what the patient said to you. Please also include any thoughts/feelings you think this patient had, that were not expressed to you.

Once completed, say: Please can you read out what you have written?

If not explored: Generally how do you feel about this patient during the consultation?

Probe:

- Concerned or relaxed?
- Frustrated or satisfied?
- Why do you have these feelings?

If not explored: To what extent, did you inform this patient that they are uncontrolled/not reaching goal ?

- How did you explain this to them? Exactly what words did you use?
- How did this patient react? What did they say to you?
- How do you think this patient felt?

If Doctor did not inform patient that they are uncontrolled ask:

- Why did you not inform this patient they are uncontrolled/not reaching goal? Please explain fully
    - Have you informed the patient he/she is uncontrolled during prior consultations?
        - If so, when?
        - What was discussed?
- How typical is this with you other uncontrolled patients? Please explain fully.

If not explored: How do you think your patient felt?

- Why do you think they felt this way?
- Do you think they had any questions or concerns that they did not raise with you?
    - If, so what?
    - Why do you think they do not discuss these questions/concerns with

you?

If not explored: Did you discuss making a treatment adjustment with this patient? Why/Why not?

- **If switching not discussed:** Do you think you will switch this patient's treatment at some point? Why/Why not?
    - When?
    - To which treatment? Why?

Did any family members or other caregivers accompany this patient?

- Who where they?
- What did they say to you/the patient?
- Do you prefer patients attending their consultation with family member/caregiver or do you prefer they attend on their own?
    - o How does this differ by patient type?
- To what extent, if at all, do you think the family/patient care givers play an important role in helping the patient to reach goal?

I would now like to discuss your interactions with the next patient profile (self completion sheet 1). Please can you complete the another self completion sheet 3 , however this time please record only the thoughts and verbal interactions that were <u>different</u> from the patient we just discussed.

Moderator: If easier/quicker, please use self completion 3 as a visual stimulus for the respondent to verbally express the patient/doctor interaction (instead of them writing/completing the sheet)

Hand new/blank version of self completion 3 to respondent

Once completed, say: Please can you read out what you have written?

How was your interaction different with this patient compared to the patient (s) we previously discussed?

Probe differences in terms of:

- What you and this patient discussed?
- What the doctor felt about this patient?

If not explored: Did you discuss making a treatment adjustment with this patient? Why/Why not?

- **If switching not discussed:** Do you think you will switch this patient's treatment at some point? Why/Why not?

- When?
- To which treatment? Why?

Repeat self completion 3 exercise and above probes for remaining patient profile(s)

Section 4 - Uncontrolled hypertension: Satisfaction and unmet needs (8 mins)

Objective: Exploration of satisfaction with managing/treating uncontrolled hypertension and identification of unmet needs and key areas for improvement

19) How satisfied are you with the treatment and management of uncontrolled hypertension patients?
    - What improvements, if any, do you think are needed?

20) What, if anything, could be done to get more patients to reach goal? Please explain fully.

21) What improvements, if any, would you like to make to pharmacological agents to ensure better control?
    - How satisfied/frustrated are they with the options that are available to treat patients?
    - **If not discussed:** What improvements would you like to make to ARBs to ensure better control?
        - o How does this differ by ARB brand?

22) How can the patient's attitude and behavior be changed to improve control?

    Probe:
    - Lifestyle modifications (i.e. healthier diet, exercise etc)?
    - Greater adherence to medication?
        - o Overall, how satisfied are you with patient compliance to their medication?

Section 5 – Product Profile Assessment (15 mins)

Objective: Detailed evaluation of product X and identification of suited patient types and product positioning

For the last section of this interview I would like to discuss in detail a new hypertension pharmacological agent.

23) Before I show the product profile, can you tell me if you are aware of any new products in development for hypertensions?

- Please tell me what you know about this new product (s)?

Hand Product Profile X

24) What do you think about product X?

25) What are the benefits/advantages?

- **Probe:** What are your thoughts regarding product X's superior blood pressure lowering benefit?
    - o To what extent, if at all, will this benefit your current treatment approach?
- **Probe**: What are your thoughts on product X being a "once daily" pill?
    - o To what extent, if at all, is this a benefit?

Probe: What are you thoughts on product X offering 24 hour protection?

To what extent, if at all, is this a benefit?

To what extent, if at all, is the ability to take the pill at any time of the day or night a benefit to your patients? Why?

To what extent, if at all, does this differ from current ARBs?

26) What are drawbacks/disadvantages?

27) I would now like you to imagine that product X has been launched in your market and available in this market for at least one year.

On a scale of 1 to 10, where 1 is unlikely and 10 is very likely, how likely would you be to prescribe product X?

- Please explain your rating

28) In what patient types do you think you would prescribe product X to?

- Why these patients?
- What drugs are they currently on?

Do anticipate switching existing patients to Product X or adding Product X to their existing regimen? Please explain fully.

29) What patient types would you definitely not consider prescribing product X to?

- What type of patients are they?
- What treatments are these patients currently receiving?
- Why do you think these patients are not suitable for product X?

If not already explored: How would product X impact your current ARB use?

- Could product X replace any of the current ARBs you use? Why/why not?
- **If not mentioned:** To what extent, has the superior blood pressure lowering benefit impacted your decision to switch/not to switch to product X?

30) Please refer to the patient types you mentioned earlier in self completion sheet 1.

- Do you think you would switch any of these patients from their current treatment to product X?
    - Why/why not?
- Do you think you would add product X to any of their current treatment?
    - Why/why not?

Interviewer show product profile A – showcard 2

- **Please review this patient profile**
    - To what extent would you prescribe product X to this patient type?
    - Why/why not?
    - What proportion of your uncontrolled patients does this patient represent?
    - What would be your current treatment approach to this patient?
    - To what extent, if at all, would you switch this current approach to product X? Why/why not?

Ask permission to recontact respondent if need to clarify any points with them via a short telephone call.

Thank & Close

Self Completion Sheet 1

ARB Brand	Proportion of ARB patients receiving brand	Typical Dose	Typical duration of treatment

Self Completion Sheet 2

Self Completion 3

Uncontrolled Hypertension Patient Profile A		
Gender	Male	Female
Age		
Occupation level (if you know)		
Co Morbidities		
Years/months since diagnosis with		

hypertension	
Years/months since having uncontrolled hypertension	
Current hypertension treatment Approach	

**Uncontrolled Hypertension Patient Profile B**		
Gender	Male	Female
Age		
Occupation level (if you know)		
Co Morbidities		
Years/months since diagnosis with hypertension		

Years/months since having uncontrolled hypertension	
Current hypertension treatment Approach	

Uncontrolled Hypertension Patient Profile C		
Gender	Male	Female
Age		
Occupation level (if you know)		
Co Morbidities		
Years/months since diagnosis with		

hypertension	
Years/months since having uncontrolled hypertension	
Current hypertension treatment Approach	
